# Does the therapeutic alliance process explain the results of the Egokitzen post-divorce intervention program?

**DOI:** 10.3389/fpsyg.2024.1419968

**Published:** 2025-01-23

**Authors:** Irati Alvarez, Marta Herrero, Ana Martínez-Pampliega

**Affiliations:** ^1^Begoñako Andra Mari Teacher Training University College, BAM, Derio, Biscay, Spain; ^2^Department of Psychology, Health Sciences Faculty, University of Deusto, Bilbao, Spain

**Keywords:** therapeutic alliance, emotion regulation, symptomatology, divorce, group

## Abstract

**Objective:**

The objective of this study was to explain the relationship between therapeutic alliance and the changes observed in the parents’ psychological symptomatology after participation in the Egokitzen program, analyzing the mediating role of emotion regulation.

**Methods:**

The study involved 117 divorced parents and 40 therapists.

**Results:**

It has been observed that the early development and maintenance of the therapeutic alliance influence the parents’ psychological symptomatology after the intervention, through emotion regulation.

**Conclusion:**

The study reinforce the role of the therapeutic alliance as a determining factor in the success of group interventions. This effect has turned out to be indirect through emotion regulation, highlighting the importance of emotional management.

## Introduction

Nowadays, destructive divorce is considered a complex, stressful, and emotionally very intense process (Amato and Hohmann-Marriott, 2007; Bodenmann et al., 2007), with repercussions in the mental health of the people who face it (Kiecolt-Glaser, 2018; Sbarra, 2015; Stack and Scourfield, 2015; Zulkarnain and Korenman, 2019). Among the consequences of this process, the literature has emphasized its impact at an emotional level as well as psychological symptomatology (Braver et al., 2016; Sandler et al., 2020), especially of a depressive type (Stack and Scourfield, 2015; Zulkarnain and Korenman, 2019).

Given the significant impact of destructive divorce on mental health, over the years, many group intervention programs have emerged that address emotion regulation and the associated symptomatology to facilitate the process of adaption of the people involved in this process (Malcore et al., 2010; Vélez et al., 2012). The objectives of these preventive programs include: (a) generating an environment of support that encourages the cathartic expression of the experiences concerning divorce, (b) providing the opportunity to solve problems and develop coping skills that help them manage their emotions, (c) relieving the stress arising from separation, and (d) developing the process of breaking the emotional bond with the ex-partner (Blaisure and Geasler, 2005; Geasler and Blaisure, 1999; Grych, 2005; Pedro-Carroll and Jones, 2005; Wolchik et al., 2000).

In recent years, studies at the international level on the effectiveness of these programs have proliferated (Becher et al., 2018; Braver et al., 2016; Jewell et al., 2017; McIntosh and Tan, 2017; Philip and O’Brien, 2017) although, in Spain, they remain scarce, with the sole exception of the Egokitzen program (Martínez-Pampliega et al., 2015, 2021). This interest has not been linked exclusively to preventive programs but also to psychotherapeutic intervention in general.

Despite progress in the verification of its effectiveness, research is still far from knowing its explanatory mechanisms. One of the factors linked to the effectiveness that has generated the most interest, regardless of the modality of intervention, is the therapeutic alliance (Friedlander et al., 2011; Horvath et al., 2011). This interest is attested by recent meta-analyses that have gathered extensive evidence of the influence of the therapeutic alliance in therapeutic success (Flückiger et al., 2018; Friedlander et al., 2018; Karver et al., 2018). Specifically, it is suggested that the therapeutic alliance explains between 7 and 21% of therapeutic change (Crits-Christoph et al., 2011; Flückiger et al., 2018; Karver et al., 2018; Wampold and Imel, 2015; Welmers-Van de Poll et al., 2018).

## Therapeutic alliance and emotion regulation

Therapeutic alliance refers to the collaborative relationship established between client and therapist (Bordin, 1979). This conceptualization has three main components: the link between therapist and client, mutual agreement on treatment goals, and mutual agreement on the tasks necessary to achieve the established goals (Bordin, 1979).

To date, many investigations have identified the relevance of establishing a strong alliance in the first sessions of psychological treatment (Wampold and Imel, 2015; Yoo et al., 2016) and of maintaining this alliance during the therapeutic process for its good prognosis (Glebova et al., 2011; Nissen-Lie et al., 2015; Wampold and Imel, 2015). Establishing a strong therapeutic alliance allows the therapeutic context to be experienced as a safe space in which an emotional connection is established between the client and the therapist (Escudero and Friedlander, 2017; Günther-Bel et al., 2021). In addition, a strong therapeutic alliance provides a feeling of connection with the therapeutic process and unity between the client and the therapy (Escudero and Friedlander, 2017; Friedlander et al., 2006b; Friedlander et al., 2006a). By achieving a context of trust and safety, the therapist will be able to confront the client to produce greater therapeutic change (Wampold and Imel, 2015).

The specific mechanisms through which the therapeutic alliance influences the effectiveness of interventions are not yet clear. In this sense, there is emerging evidence of associations between the therapeutic alliance and emotion regulation (Owens et al., 2013; Ronningstam, 2017; Whitehead et al., 2019). Higher levels of therapeutic alliance are associated with lower levels of difficulties in regulating emotions (Burt, 2013; Knerr et al., 2011; Owens et al., 2013; Whitehead et al., 2019), understanding emotion regulation as the process through which individuals modulate their emotions and modify their behavior to achieve goals, adapt to the context or promote their well-being (Gross, 2015). Therefore, the establishment of a strong therapeutic alliance could enhance their ability to regulate emotions.

In turn, the existing literature indicates that people’s emotion regulation is related to their symptomatology (Estévez Gutiérrez et al., 2014; Garnefski and Kraaij, 2006). That is, those with greater abilities to regulate emotions suffer lower levels of symptomatology (Gross and Feldman Barrett, 2011). Recently, Fisher et al. (2016) integrated both emotion regulation and the therapeutic alliance in their study and identified the important role of both variables as determinants of the therapeutic process and the prediction of the clients’ functioning.

### The present study

The review of the literature has highlighted the need to understand the effectiveness of post-divorce intervention programs has been identified, with divorce being regarded as an emotionally very intense process. Understanding the effectiveness of post-divorce group interventions could benefit from deepening the therapeutic alliance and its impact on emotion regulation. To date, we know of no studies in this regard.

This study is proposed to analyze, through a longitudinal study, the development of the alliance throughout the implementation of a post-divorce intervention program, and to deepen the relationship of the alliance with parents’ emotion regulation and symptomatology. The program implemented will be the Egokitzen program, which, as indicated, is the only one that currently has studies of efficacy and effectiveness in Spain (Martínez-Pampliega et al., 2015, 2021). The data of the therapeutic alliance will be collected from the therapist’s perception, because it is more related to the outcome of the therapy than the client’s perception (Baldwin et al., 2007; Culina et al., 2023; Del Re et al., 2012).

### Research question

Does the evolution of the therapeutic alliance in the course of therapy explain the direct change in emotion regulation and the indirect change in the parents’ psychological symptomatology?

### Hypothesis

The early development of the therapeutic alliance and its maintenance throughout the intervention will be associated with a reduction of the parents’ symptomatology, through its relationship with the parents’ increased emotion regulation.

## Methods

### Participants

The final sample was made up of 117 parents average aged 41.88 years (*SD* = 6.30). Of these, 36% were fathers and 64% mothers. These parents had on average 1.57 children (*SD* = 0.71) with an average age of 9.00 years (*SD* = 4.47). Forty-six percent of the participants had been divorced for more than 3 years, 13% from 2 to 3 years, 20% from 1 to 2 years, 9% from 6 months to 1 year, 10% from 2 to 6 months, and 2% less than 2 months. With regard to the level of education, 35% reported having primary studies, 34% high school or vocational training, 11% a middle career, 17% a higher career, and 3% a master’s degree or Ph.D.

The parents participated in 34 intervention groups supervised by two therapists. This research involved 40 therapists. The average age of the pairs of therapists was 43.43 years (*SD* = 8.88), and their professional experience was 16.84 years (*SD* = 5.72). Thirty-six percent of the pairs of therapists were made up of one man and one woman, 59% of the pairs were two women, and 5% of the pairs of therapists were made up of two men.

### Intervention

The Egokitzen post-divorce intervention program (Martínez-Pampliega et al., 2015, 2021), developed from a systemic approach to family functioning, was implemented. It is aimed at minimizing the impact of interparental conflict and building the resilience of the participants and their children. It consists of 10 sessions of 90 min (plus a previous framing session), implemented on a weekly basis, and structured around divorce and its impact, interparental conflict, and parenting. Special emphasis is placed on the emotional impact of the breakup, helping the participants to better manage their emotions. The sessions are designed to actively engage the participants through role-playing, debates, and group activities.

### Client measures

#### Emotion regulation

Emotion regulation was measured through the Spanish adaptation of the *Difficulties in Emotion Regulation Scale* (DERS; Gratz and Roemer, 2004) of Hervás and Jódar (2008). This scale examines the difficulties that can appear in the process of emotion regulation and is composed of 25 five-point Likert-type items ranging from 1 (*almost never*) to 5 (*almost always*), grouped into five dimensions: Non-Acceptance, Lack of Objectives, Impulsivity, Lack of Strategies, and Lack of Clarity. The internal consistency of the global scale was 0.95 at pre- and posttreatment. Cronbach’s alpha index was adequate in all the dimensions both at pre- and posttreatment (Non-acceptance: 0.87 and 0.93; Lack of clear objectives: 0.90 and 0.84; Impulsivity: 0.84 and 0.78; Lack of strategies: 0.91 and 0.89; Lack of Clarity: 0.71 and 0.67, respectively).

#### Parental psychological symptomatology

Psychological symptomatology was measured with the adaptation and validation in Spanish of the *Symptom Checklist-90* (SCL-90; Derogatis et al., 1973) of González de Rivera et al. (2002). The scale has 44 Likert-like items rated from 1 (*nothing or not at all*) to 4 (*very much or extremely*), with a global score contemplating Interpersonal Sensitivity, Depression, Anxiety, and Somatization. The Cronbach alpha in this study was 0.96 at the pre- and post-treatment.

### Therapists’ measures

#### Therapeutic alliance

Therapeutic alliance was measured through the *System for Observing Family Therapy Alliances* (SOFTA-s: Alvarez et al., 2020). It consists of 12 Likert-type items, ranging from 1 (*not at all*) to 5 (*very much*) grouped into four dimensions: Engaging in the therapeutic process, Emotional Connection with the therapist, Safety within the therapeutic system, and Sense of Sharing the purpose in the family. The questionnaire is completed via the therapists’ perception of the therapeutic alliance with the group. The reliability of the scale in the third session was 0.65, in the sixth session, it was 0.76, and in the ninth session, it was 0.84. So, the overall mean of the scale’s reliability was 0.75.

### Procedure

This longitudinal study was developed at 12 family visitation centers nationwide. We contacted 1,538 people to ask them if they were interested in participating in the intervention program, of whom 428 reported being interested and were summoned to a personal interview. This personal interview was attended by 360 people, who were informed about the program and who signed the informed consent. However, 107 could not meet the conditions of participation and be included in the experimental group (due to working hours, shift work, family conciliation, etc.). Finally, 117 parents completed the questionnaires on the variables of this study. They are divorced individuals users of family visitation centers.

The 40 participating therapists were formed by the members of the research team in both the evaluation and implementation of the intervention program. Each intervention group was led by two professionals, with at least one being a psychologist. The average of participants in the groups was 3.47.

Regarding data collection, to measure therapeutic change, the participants completed the DERS and SCL-90 instruments individually before the intervention group began and again at the end. The therapists, meanwhile, completed the Therapeutic Partnership Questionnaire (SOFTA-s) at the end of sessions 3, 6, and 9. The literature has shown the need to collect therapeutic alliance measurements at various times throughout the treatment in order to explain the outcome of an intervention (Crits-Christoph et al., 2011).

Participation in the investigation was voluntary and participants were ensured about the anonymity of the responses to the questionnaires. Participants were also informed of the possibility of dropping out of the investigation if they wished to do so. The investigation was approved by the ethics committee of the University of Deusto (ETK-7/16–17).

### Data analysis

The hypothesis was tested using growth curve analysis in a structural equation (SEM) framework with Mplus 7.0 (Muthén and Muthén, 2012) with the maximum likelihood estimator. Following the indications of Wang and Wang (2012), we began testing the growth model of the therapeutic alliance. In this sense, growth curve analyses allow SEM to be applied to longitudinal data analysis with repeated measures for the same subjects over time. As the therapeutic alliance was measured at three moments (i.e., in the third, sixth, and ninth sessions), two models were compared based on the possible slopes (the maximum degree of the polynomial cannot exceed the number of time points – 1 = 3–1 = 2): the linear slope model and the quadratic slope model. In both cases, to facilitate interpretation, the first measure (i.e., the third session) was set to zero as the centering point. In this way, we compared whether the therapeutic alliance had a linear or curved evolution from the beginning to the end of the intervention. At the same time, the intercept was modeled, fixing the first collected value (third session) to facilitate interpretation. Therefore, the intercept can be understood as the initial level of the therapeutic alliance.

After analyzing the growth model of the therapeutic alliance, we tested the complete model, which included the intercept and the slope of the therapeutic alliance as independent variables, changes in emotion regulation difficulties as a mediator, and changes in symptomatology as the dependent variable. For this purpose, the changes were computed as differential variables in which the pre-intervention value was subtracted from the post-intervention value to represent the changes over the course of the program. In addition, gender, age, and intervention group were included as control variables in the model.

To assess the level of fit of the model, the following goodness-of-fit indicators were considered: non-significant chi-square (*χ*^2^), Comparative Fit Index (CFI), and Tucker-Lewis Index (TLI) greater than 0.90, and Root Mean Squared Error of Approximation (RMSEA) and Standardized Root Mean Square Residual (SRMR) below 0.08 (Hu and Bentler, 1999).

## Results

First, we calculated the descriptive statistics of the observed variables of the study shown in Table 1. Secondly, the models were tested according to the hypothesis, starting by analyzing the evolution of the alliance throughout the intervention sessions.

**Table 1:** Descriptive statistics.

	Pre		Session 3		Session 6		Session 9		Post	
Study variable	*M*	SD	*M*	SD	*M*	SD	*M*	SD	*M*	SD
Therapeutic alliance			4.23	0.26	4.31	0.33	4.41	0.35		
Emotion regulation difficulties										
Non-acceptance	10.43	4.99							9.30	4.82
Lack of clear objectives	7.46	3.67							6.90	2.93
Impulsivity	7.82	3.46							7.14	2.86
Lack of strategies	11.63	5.36							11.03	4.95
Lack of clarity	7.24	3.03							7.04	2.78
Psychological symptomatology	41.07	31.61							32.42	27.70

The results of the hypothesized quadratic model led to estimation warnings that indicated problems in the specification of the model. Therefore, the quadratic function was considered inappropriate for modeling the growth curve of the therapeutic alliance (Wang and Wang, 2012). Based on this, the model was tested with the linear slope, which showed a good fit to the data, (*χ*^2^[1] = 1.50, *p* = 0.221, CFI = 1.00, TLI = 0.99, RMSEA = 0.055, SRMR = 0.132), so it was established as a growth model of therapeutic alliance. This model indicated that both the mean (*M* = 4.24, *SE* = 0.02, *p* < 0.001) and the variance (*σ* = 0.05, *SE* = 0.01, *p* < 0.001) of the intercept were significant, yielding significant differences in the initial levels of therapeutic alliance among participants. Also, the mean (*M* = 0.08, *SE* = 0.03, *p* = 0.004) and variance (σ = 0.02, *SE* = 0.01, *p* = 0.006) of the linear slope were significant. We therefore note that the therapeutic alliance tended to increase in a linear and non-quadratic way throughout the intervention process, and that the participants differed significantly in the increase of alliance across the program sessions.

From the growth model of therapeutic alliance, we tested the final model, including the change in the difficulties of emotion regulation as a mediator, the change in psychological symptomatology as a dependent variable, and the control variables. This final model showed a good fit to the data (*χ*^2^[45] = 58.33, *p* = 0.087, CFI = 0.95, TLI = 0.94, RMSEA = 0.050, SRMR = 0.070). The correlations between the model variables are shown in Table 2.

**Table 2:** Bivariate correlations of the model variables.

	Correlations				
Variables	1	2	3	4	5
1. Gender					
2. Age	−0.04				
Therapeutic alliance					
3. Intercept	< 0.01	< 0.01			
4. Slope	< 0.01	< 0.01	< 0.01		
5. ∆ Emotion regulation difficulties	−0.01	−0.01	−0.35***	0.03	
6. ∆ Psychological symptomatology	−0.18	0.04	−0.15	0.03	0.35***

*** *p* < 0.001.

**Figure 1:**
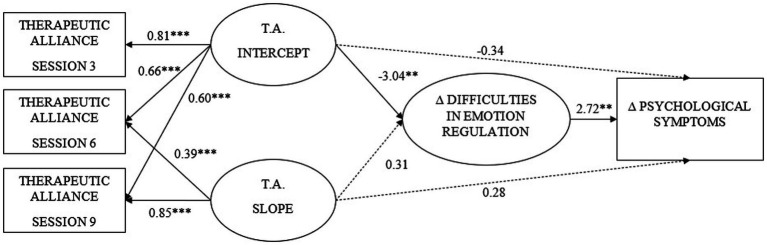
Standardized growth model coefficients. TA, Therapeutic alliance. Dashed lines represent non-significant coefficients. **p < 0.01, *** *p* < 0.001.

As shown in Figure 1, the results of the final model indicated that the intercept of therapeutic alliance was significantly and negatively related to the increase in emotion regulation difficulties, but the slope of the therapeutic alliance showed a nonsignificant relationship with the changes in emotion regulation difficulties. Therefore, higher levels of therapeutic alliance, and not its increase across the sessions, were related to the decrease in emotion regulation difficulties from pre- to postintervention.

With regard to changes in symptomatology, we noted that the therapeutic alliance showed no significant direct relationship either with the intercept or with the slope. However, changes in the difficulty of emotion regulation showed a significant and positive relationship with changes in symptomatology. Thus, those who experienced a reduction in their emotion regulation difficulties tended to reduce their symptomatology.

Finally, the indirect effect of the intercept of the therapeutic alliance on symptomatology was analyzed, finding that it was significant (−10.07, *SE* = 5.14, *p* = 0.050), such that higher levels of therapeutic alliance as of the first sessions were related to a greater reduction of symptomatology due to the effect on emotion regulation difficulties. This model explained 18% of the variance of emotion regulation difficulties and 15% of psychological symptomatology.

## Discussion

The objective of this study was to explain the relationship between therapeutic alliance and the changes observed in the parents’ psychological symptomatology after participation in the Egokitzen program, analyzing the mediating role of emotion regulation. The results support the hypothesis: it has been observed that the early development and maintenance of the therapeutic alliance influence the parents’ psychological symptomatology after the intervention, through emotion regulation.

With regard to the research question focused on the role of the therapeutic alliance, we can highlight two aspects: (1) we found that a strong construction of the therapeutic alliance at the beginning of the intervention, together with its maintenance throughout the treatment, explains the change in the parents’ symptomatology; (2) this impact on symptomatology occurred through the observed change in the parents’ emotion regulation. These two points will be addressed in more detail below.

On the one hand, the findings of this study confirm the importance of building a strong therapeutic alliance in the first sessions of treatment and maintaining it during the process to achieve therapeutic change. These results are relevant because, in this area of research, there is some controversy about the best trajectory of the therapeutic alliance to achieve therapeutic success. In this regard, the study provides additional data to the evidence provided by Nissen-Lie et al. (2015) or Yoo et al. (2016), among others, compared to other studies (Chu et al., 2014; Escudero et al., 2022; Schmidt et al., 2023) that, on the contrary, found support for the increase of the therapeutic alliance throughout the intervention as a more favorable condition to obtain better therapeutic results. Although in clinical practice, it is a challenge for therapists to maintain a stable therapeutic alliance during the intervention and avoid breakdowns in it, the results obtained highlight the special care that therapists must exert. Finding strategies to achieve this may have to be the goal. In this sense, the studies of Aron (2006) and Larsson et al. (2018) directed attention toward meta-communication about disagreements and impasses in the therapeutic relationship, that is, how to take advantage of breakdowns in the therapeutic alliance and turn them into opportunities for the benefit of the therapeutic process.

On the other hand, our research has helped to clarify the mechanisms through which the therapeutic alliance can be related to the success of interventions, as measured in this study through the parents’ symptomatology. Specifically, we identified emotion regulation as a mediating variable. Although this variable had shown its relevance in other contexts (Fresco et al., 2013; Peña-Sarrionandia et al., 2015), no research had been developed till now that reflected its importance in preventive post-divorce intervention programs. These results seem to be consistent with the literature carried out in clinical context, which has emphasized the relationship between therapist and client as a corrective emotional experience (Alexander and French, 1946; Castonguay and Hill, 2012; Safran and Muran, 2000), allowing clients to acquire better management of their emotions throughout the intervention, and reducing the associated psychological symptomatology. Despite the promising results obtained, the specificity of the intervention in this study will require further research to analyze the role of emotion regulation in other therapeutic modalities.

In this sense, we emphasize the fact that this research has identified the relevance of the therapeutic alliance in a group context, a modality scarcely researched so far, identifying its role in the therapeutic success, as in other modalities (Baldwin et al., 2007; Del Re et al., 2012; Nissen-Lie et al., 2015). The results of the study allow us to affirm the importance of the therapists’ directing their efforts in the first sessions to achieve therapeutic engagement and working in collaboration with the members of the group to achieve the objectives, also in group interventions and even preventive interventions.

Finally, the study has provided support to those researchers who have emphasized the need to address the design, analyzing the therapeutic alliance process through different sessions, to better understand its relationship with the results of the therapeutic interventions (Sexton et al., 2004). In fact, it should be noted that this research, with three moments of measurement of the therapeutic alliance, has allowed us to explain a fairly acceptable percentage (15–18%) of therapeutic change (Crits-Christoph et al., 2011; Flückiger et al., 2018; Karver et al., 2018; Wampold and Imel, 2015; Welmers-Van de Poll et al., 2018).

However, this study has several limitations that suggest a cautious interpretation of the results. First, the therapist sample is small. While this is explained by the specificity of the context and the intervention model. A greater number would favor a greater generalization of the findings obtained. Another limitation of the study is the self-reported nature of the measures instead of observational measures, or, from the point of view of the design, the absence of relevant follow-up measures to understand causal relationships. In this sense, it would be important to collect data throughout more time periods, and longer periods, in order to know if the effect persists in the long term. It would also be relevant for future research, to consider additional variables such as the therapists’ characteristics (e.g., personality), aspects that have not yet been addressed in post-divorce group interventions or differences between divorced individuals (e. g., level of conflict).

In short, to our knowledge, this research is the first study that addresses the explanatory mechanisms of the therapeutic alliance in a group context with post-divorce interventions. The study has helped to reinforce the role of the therapeutic alliance as a determining factor in the success of group interventions, because of its relationship with the participants’ symptomatology. This effect has turned out to be indirect through emotion regulation, highlighting the importance of managing emotional processes for therapeutic success, also in post-divorce group interventions. The generalization of this result to other modalities and therapeutic objectives remains to be analyzed.

## Data Availability

The raw data supporting the conclusions of this article will be made available by the authors, without undue reservation.
